# Supplementary material for: The effect of COVID-19 on public hospital revenues in Iran: An interrupted time-series analysis
Source: PLoS One. 2022 Mar 31;17(3):e0266343. doi: 10.1371/journal.pone.0266343 (PMC8970352; doi:10.1371/journal.pone.0266343)
Supplement: Attachment — Submitted filename: Response to Reviewers.docx [file pone.0266343.s002.docx]

Reviewer #1: Abstract

1. Please explain more about data collection in the method section.

Thanks for your comment. We added.
2. Please delete million in the ($172,636 million) and replace with (thousand).

Thanks for your comment. We added.
3. Please delete the word (dramatically) in the conclusion section.

Thanks for your comment. Done.
4. Please replace (covid-19, hospital, health financing, revenue, interrupted time series analysis, health policy, Iran) with old keywords.

Thanks for your comment. Done.

Main text:
1. Please update the incidence and mortality rate in the introduction section.

Thanks for your comment. We added.

2. Please more explain the difference between services related to the public and private hospitals in Iran.

Thanks for your comment. Done.
3. Please mention ethical approval in the method section.

Thanks for your comment. Done.

4. Please explain sensitivity analysis in the results section.

Thanks for your comment. Done.

5. Figure 3 is low quality, please revise and promote the quality.

Thanks for your comment. We added a high-quality figure.

6. Please explain more about limitations in the discussion section.

Thanks for your comment. Done.

7. Please explain more about tables in the text in the results section.

Thanks for your comment. Done.

Reviewer #2:

A) The delete the (pandemic, Lorestan and insights from an) in the Title of the manuscript.

Thanks for your comment. Done.

B) I think the method section can be improved in the abstract section.

Thanks for your comment. Done.

C) What was the role of Iranian government assistance to hospitals?. Add sentences in the introduction section.

Thanks for your comment. Done.

D) Explain how to collect hospital revenue in the method section.

Thanks for your comment. Done.
